# Supplementary material for: Machine Learning Algorithms to Predict Mortality of Neonates on Mechanical Intubation for Respiratory Failure
Source: Biomedicines. 2021 Oct 2;9(10):1377. doi: 10.3390/biomedicines9101377 (PMC8533201; doi:10.3390/biomedicines9101377)
Supplement: Supplementary file 1 [file biomedicines-09-01377-s001.zip › biomedicines-1373742-supplementary.pdf]

biomedicines

MDPI

Article

Machine Learning Algorithms to Predict Mortality of Neonates on Mechanical Intubation for Respiratory Failure

Supplementary materials

Supplementary Figure 1.

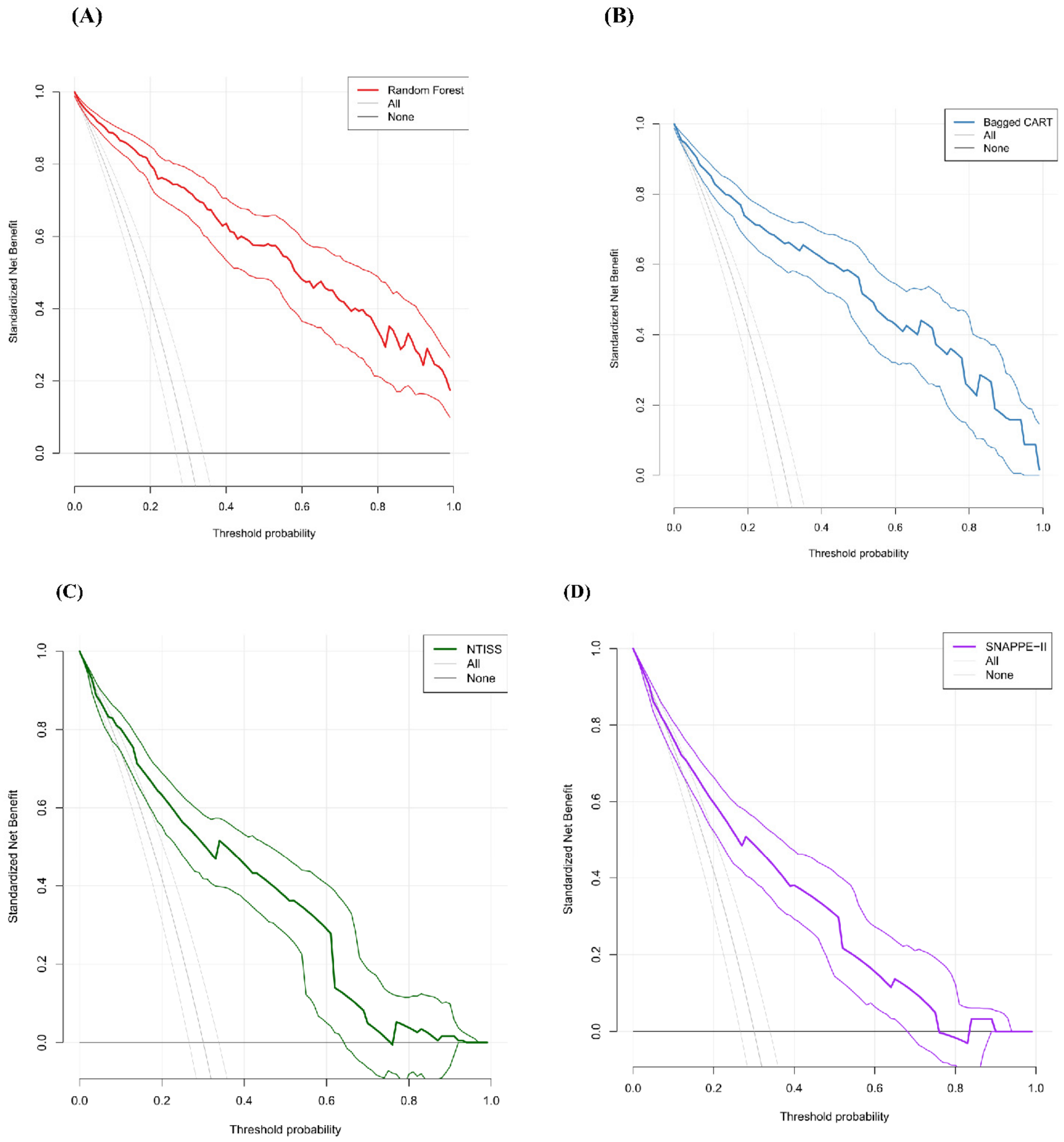

(E)

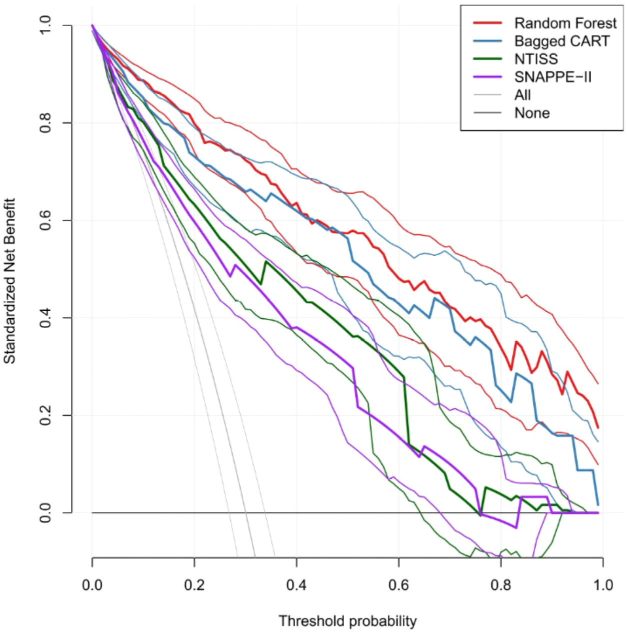

Supplementary Figure 1. Decision curve analysis for predicting NICU mortality in the test. (A) Random forest; (B) Bagged CART; (C) NTISS; (D) SNAPPE-II; (E) Total

Figure S1. Decision curve analysis for predicting NICU mortality in the test. (**A**) Random forest; (**B**) Bagged CART; (**C**) NTISS; (**D**) SNAPPE-II; (**E**) Total.

Table S1. Patients characteristics of the survivors versus the NICU mortality.

Characteristics	All study subjects (total n = 1734)	NICU survivors (total n = 1456)	NICU mortality (total n = 278)	P values
Cases demographics				
Gestational age (weeks), median (IQR)	27.0 (25.0-31.3)	27.4 (25.5-31.5)	26.3 (24.5-31.0)	< 0.001
Birth weight (g), median (IQR)	915.0 (703.5-1480.0)	960.0 (760.0-1500.0)	785.0 (620.0-1335.0)	< 0.001
Gender (male), n (%)	1029 (59.3)	875 (60.1)	154 (55.4)	0.049
Birth by NSD/Cesarean section, n (%)	548 (31.6)/1186 (68.4)	458 (31.5)/998 (68.5)	90 (32.4)/188 (67.6)	0.488
5 minutes Apgar score < 7, n (%)	566 (32.6)	440 (30.2)	126 (45.3)	< 0.001
Inborn/outborn, n (%)	1365 (78.7)/369 (21.3)	1145 (78.6)/311 (21.4)	220 (79.1)/58 (20.9)	0.895
Premature rupture of membrane, n (%)	530 (30.6)	450 (30.9)	80 (28.8)	0.268
Perinatal asphyxia, n (%)	354 (20.4)	268 (18.4)	86 (30.9)	< 0.001
Onset of respiratory failure, day (median [IQR])	1.0 (1.0-3.0)	1.0 (1.0-3.0)	1.0 (1.0-2.0)	0.147
Disease entities of respiratory failure, n (%)				
Respiratory distress syndrome (≥ Gr II)	1047 (60.3)	897 (61.6)	150 (54.0)	0.001
Transient tachypnea of newborn	83 (4.8)	73 (5.0)	10 (3.6)	0.258
Complicated cardiovascular diseases	28 (1.6)	16 (1.1)	12 (4.3)	0.001
Symptomatic patent ductus arteriosus	662 (38.2)	584 (40.1)	78 (28.5)	< 0.001

Persistent pulmonary hypertension of newborn	278 (16.0)	201 (13.8)	77 (27.7)	< 0.001
Pulmonary hemorrhage	120 (6.9)	66 (4.5)	54 (19.4)	< 0.001
Pneumonia	85 (4.9)	68 (4.7)	17 (6.1)	0.384
Air leak syndrome[&]	188 (10.8)	146 (10.0)	42 (15.1)	0.038
Meconium aspiration syndrome	50 (2.9)	47 (3.2)	3 (1.1)	0.003
Sepsis	271 (15.6)	202 (13.9)	69 (24.8)	< 0.001
Hydrops fetalis	34 (2.0)	25 (1.7)	9 (3.2)	0.145
Others[#]	26 (1.5)	20 (1.4)	6 (2.2)	0.267
Presences of any chronic comorbidities, n (%)	379 (21.8)	248 (17.0)	131 (47.1)	< 0.001
Presences of central venous catheter, n (%)	522 (30.1)	349 (24.0)	173 (62.2)	< 0.001
Initial ventilator requirement*, n (%)				< 0.001
Intubation with mechanical ventilation	1168 (67.4)	1034 (71.0)	134 (48.2)	
Initial $FiO_2 \leq 50$	671 (38.7)	603 (41.4)	68 (24.5)	
Initial $FiO_2 > 50$	497 (28.7)	431 (29.6)	66 (23.7)	
On high frequency oscillatory ventilation	566 (32.6)	422 (29.0)	144 (51.8)	
High setting ($FiO_2 \leq 50$)	248 (14.3)	184 (12.6)	64 (23.0)	
Low setting ($FiO_2 > 50$)	318 (18.3)	238 (16.3)	80 (28.8)	
Oxygenation index, median (IQR)	11.0 (6.0-20.0)	9.0 (5.0-16.0)	18.0 (9.0-34.0)	< 0.001
$AaDO_2$, median (IQR)	273.0 (166.0-478.0)	239.0 (148.0-410.0)	453.0 (233.0-568.0)	< 0.001
Use of iNO	285 (16.4)	164 (13.0)	121 (25.4)	< 0.001
Clinical features*, n (%)				
Intravascular volume expansion	1415 (81.6)	1160 (79.7)	255 (91.7)	< 0.001
Requirement of cardiac inotropic agents	1206 (69.6)	978 (67.2)	228 (82.0)	< 0.001
Metabolic acidosis	677 (39.0)	525 (36.1)	152 (54.8)	< 0.001
Coagulopathy	1226 (70.7)	1002 (68.8)	224 (80.1)	< 0.001
Requirement of blood transfusion**	558 (32.2)	428 (29.4)	130 (46.8)	< 0.001
Laboratory data at onset of respiratory failure				
Leukocytosis or leukopenia	446 (25.7)	348 (23.9)	98 (35.3)	< 0.001
Shift to left in WBC (immature > 20%)	158 (9.1)	102 (7.0)	56 (20.1)	< 0.001
Anemia (hemoglobin level < 11.5 g/dL)	317 (18.3)	225 (15.4)	92 (33.1)	< 0.001
Thrombocytopenia (platelet < 150,000/ul)	434 (25.0)	314 (21.6)	120 (43.2)	< 0.001
C-reactive protein (mg/dL), median (IQR)	5.0 (2.0-19.5)	5.0 (2.0-19.0)	5.0 (2.0-20.0)	0.869
Severity score at onset of respiratory failure				
NTISS (median [IQR])	23.0 (21.0-26.0)	22.0 (19.0-25.0)	27.0 (24.0-29.0)	< 0.001
SNAPPE-II (median [IQR])	28.0 (22.0-40.0)	23.0 (18.0-36.0)	42.0 (32.0-51.0)	< 0.001

FiO_2: fraction of inspired oxygen; NSD: normal spontaneous delivery; IQR: interquartile range; iNO: inhaled nitric oxide; HFOV: high-frequency oscillatory ventilator; WBC: white blood cell; NTISS score: Neonatal Therapeutic Intervention Scoring System; SNAPPE-II: Score for Neonatal Acute Physiology Perinatal Extension II

&Including pneumothorax, pneumomediastinum and pulmonary interstitial emphysema

*At onset of respiratory failure

**Including leukocyte poor red blood cell and/or platelet transfusion

#Including congenital diapharmatic hernia (21), pulmonary sequestration (3), hydrops fetalis

Table S2. *p* values for differences between the machine learning models for NICU mortality prediction in the test set.

	**Random Forest**	**Bagged CART**	**SVM**	**E-nRR**	**KNN**	**ANN**	**XGB**
Random Forest	-	< 0.001	< 0.001	< 0.001	< 0.001	< 0.001	< 0.001
Bagged CART	< 0.001	-	< 0.001	< 0.001	< 0.001	< 0.001	< 0.001
SVM	< 0.001	< 0.001	-	0.0022	< 0.001	< 0.001	< 0.001
E-nRR	< 0.001	< 0.001	0.0022	-	0.012	< 0.001	0.008
KNN	< 0.001	< 0.001	< 0.001	0.012	-	0.429	0.385
ANN	< 0.001	< 0.001	< 0.001	< 0.001	0.429	-	0.817
XGB	< 0.001	< 0.001	< 0.001	0.008	0.385	0.817	-

ANN: artificial neural network; KNN: k-nearest neighbor; SVM: support vector machine; RF: XGB: extreme gradient boost (XGB); bagged CART: bagged classification and regression tree; E-nRR: elastic-net regularized logistic linear regression.
